# Spatial analysis of risk areas of congenital anomalies in Brazil, 2012-2021

**DOI:** 10.1590/S2237-96222025v34e20250240.en

**Published:** 2025-05-09

**Authors:** Suzana de Souza, Clarissa Gutierrez Carvalho, Lavinia Schuler-Faccini

**Affiliations:** 1 Universidade Federal do Rio Grande do Sul, Programa de Pós-graduação em Saúde da Criança e do Adolescente, Porto Alegre, RS, Brazil; 2 Secretaria Estadual de Saúde do Rio Grande do Sul, Assessoria de Gestão e Planejamento, Porto Alegre, RS, Brazil; 3 Hospital de Clínicas de Porto Alegre, Serviço de Neonatologia, Porto Alegre, RS, Brazil; 4 Hospital de Clínicas de Porto Alegre, Serviço de Genética Médica, Porto Alegre, RS, Brazil

**Keywords:** Congenital Abnormalities, Risk, Newborn, Spatial Analysis, Environment and Public Health, Anomalías Congénitas, Riesgo, Recién nacido, Análisis Espacial, Medio Ambiente y Salud Pública

## Abstract

**Objective:**

To identify areas at risk for congenital anomalies in Brazil, from 2012 to 2021.

**Method:**

Time series analysis using data from the Live Birth Information System. Prevalence of anomalies in the period was calculated according to immediate geographic region. Spatial analysis was performed by calculating the Global and Local Moran index and spatial scanning, with calculation of Relative Risk (RR) and p-value, for the risk areas.

**Results:**

Areas at higher risk for anomalies were identified in the Northeast, Southeast, and Southern regions. The Northeast region had a higher number of clusters (n=31) and higher prevalence of nervous system anomalies compared to the other regions (9.7/10,000 live births). The highest risk of anomalies compared to the other areas was found in the state of Paraíba (RR 2.4; p-value<0.001).

**Conclusion:**

Disparities in the distribution of congenital anomalies were identified in Brazil, with risk areas in the Northeast, Southeast, and Southern regions.

## Resumen

**Objetivo:**

Identificar áreas de risco de anomalias congênitas no Brasil no período 2012-2021.

**Método:**

Trata-se de análise de série temporal utilizando dados do Sistema de Informação Sobre Nascidos Vivos. A prevalência de anomalia no período foi calculada por região geográfica imediata. A análise espacial foi realizada pelo cálculo do índice de Moran global e local e varredura espacial, com cálculo do risco relativo (RR) e p-valor, para as áreas de risco.

**Resultados:**

Áreas de maior risco de anomalias foram identificadas nas regiões Nordeste, Sudeste e Sul. O Nordeste apresentou maior número de aglomerados de risco (n=31) e maior prevalência de anomalias do sistema nervoso em relação às outras regiões (9,7/10.000 nascidos vivos). O maior risco de anomalias em relação às demais áreas foi observado na Paraíba (RR 2,4; p-valor<0,001). Conclusão: Disparidades na distribuição de anomalias congênitas no Brasil foram identificadas, com áreas de risco nas regiões Nordeste, Sudeste e Sul.

## Introduction

Congenital anomaly can be defined as any structural, functional or metabolic change that affects embryonic and/or fetal development. Genetic factors, environmental factors or a combination of both (multifactorial) are contained in its etiology, although, in many cases, it is not possible to identify it (1). Globally, between 3% and 6% of children were born with some type of anomaly in 2022 (2). It was one of the leading causes of mortality and disability among children in industrialized countries (3). Following the improvement of sanitary and nutritional conditions and the reduction in child mortality due to infectious and parasitic diseases, congenital anomalies have become the leading cause of infant deaths in Brazil, accounting for 22% of deaths in children under one year old (2). 

Brazil has great genetic variability and diversity of environmental factors, including socioeconomic, cultural, racial and ethnic variables that can impact the distribution of diseases and health problems (4). Knowing how congenital anomalies are distributed spatially makes it possible to recognize clusters. This can be a starting point for identifying genetic susceptibility and environmental factors associated with occurrence of congenital anomalies in regions with high prevalence.

Primary prevention of congenital anomalies is based on environmental control of risk factors. Carrying out studies to identify these areas is of fundamental importance, since this enables health action planning to be better targeted towards modifiable risk factors. Knowing the spatial distribution of congenital anomalies can also contribute to the formulation of public policies from the perspective of tertiary prevention. This aims to avoid complications from congenital anomalies through adequate rehabilitation and correction (2). Spatial epidemiology allows an early view of collective risk. This early view is important in terms of public health, as endemic processes and public health interventions in different social groups need to be analyzed from an ecological perspective (5).

The objective of this study was to identify congenital anomaly risk areas in Brazil.

## Methods

### Design and setting

This is a time series study that took the immediate geographic regions of Brazil as its units of analysis, namely: 188 in the Northeast; 160 in the Southeast; 96 in the South; 64 in the North; and 52 in the Midwest. These regions reflect the organization of productive space, developing centers and urban-industrial structure (6). We chose this unit of analysis for this study in order to correct random fluctuations and provide better stability, especially in areas with very small populations. 

### Participants

The study population consisted of all live births with congenital anomaly registered with live birth certificates in Brazil between 2012 and 2021.

### Data source

Data on all live births, distributed across political regions and immediate geographic regions, were extracted from the Live Birth Information System (*Sistema de Informação Sobre Nascidos Vivos* - SINASC) using TabNet, available on the website of the Brazilian National Health System Information Technology Department.

### Variables

The data were extracted from the SINASC according to the type of anomaly, considering the following codes from chapter 17, Congenital malformations, deformations and chromosomal abnormalities (Q00-Q99), of the 10th version of the International Statistical Classification of Diseases and Related Health Problems: congenital malformations of the nervous system (Q00-Q07); spina bifida (Q05); congenital malformations of the circulatory system (Q20-Q28); cleft lip and cleft palate (Q35-Q37); congenital absence, atresia e stenosis of the small intestine (Q38-Q41); digestive system (Q48-Q45); undescended testicle (Q53); congenital malformations of the urinary system (Q60-Q64); congenital deformities of the hip (Q65); congenital deformities of the feet (Q66); congenital malformations and deformations of the musculoskeletal system (Q65-Q79); other congenital malformations (Q80-Q89); and chromosomal abnormalities (Q90-Q99).

### Study size and data measurement

The data were organized on an Excel spreadsheet. Prevalence was calculated according to type of congenital anomaly in the total study period (2012 to 2021) for the five regions of the country. To calculate prevalence, the sum of cases of congenital anomalies in the region formed the numerator, while the denominator was formed by the sum of the total number of live births in the same region, multiplied by 10,000. Additionally, a descriptive analysis of the percentage distribution of records was performed, according to type of anomaly and region. For this calculation, the type of congenital anomaly was taken as the numerator, while the denominator was formed by the sum of all types of anomaly, multiplied by one hundred.

### Statistical methods

For spatial statistical analysis, a thematic map of prevalence was built, categorized into distribution quintiles. The SIRGAS 2000 Datum was used as a projection system for geographic coordinates, this being a geocentric reference system for the Americas, officially adopted in Brazil (7). The maps of Brazil were obtained - in vector format (shapefile) – from the website of the Brazilian Institute of Geography and Statistics (*Instituto Brasileiro de Geografia e Estatística*) (8).

Risk areas were identified using spatial autocorrelation analysis and spatial scanning analysis. The spatial autocorrelation analysis was carried out by calculating the global Moran index (Moran’s I) and the local indicator of spatial association (LISA). “Queen” type contiguity was used as a matrix of spatial weights, which considers vertices (nodes) with point connections (9).

The Moran scatterplot was used to visualize spatial autocorrelation. The analysis was performed by comparing prevalence of anomaly in each immediate geographic region with its prevalence in neighboring regions. Data were presented in four quadrants: high-high, low-low, high-low and low-high. Data that were concentrated in quadrant 1 were considered risk clusters (high-high), where prevalence of congenital anomaly in each polygon and the average value of prevalence in neighboring polygons was higher than the global average. The diagram was built based on normalized congenital anomaly prevalence values, that is, the original values subtracted from the global mean value, divided by the standard deviation. Resulting association was represented by Moran’s I, which presents values between 0 and 1 (10).

Additionally, we used LISA to analyze local spatial association. It produced the degree of autocorrelation in each immediate geographic region and allowed a more adequate geographic visualization of the degree of concentration of congenital anomalies. The cluster map was obtained by combining information from the Moran scatterplot with LISA statistics (11).

We used the spatial scanning analysis technique (12) to identify areas at risk of congenital anomalies, by inserting a virtual circle around the center of the immediate geographic regions and calculating the prevalence of occurrence of anomalies. Risk clusters were considered to exist when the prevalence rate found was higher than expected, that is, the number of anomalies in each area was not proportional to the size of its population. 

Spatial scanning involved use of the discrete Poisson model, and the analysis was based on the total number of cases found with a maximum cluster size equal to 50% of the exposed population. The analyses were based on purely spatial variation, with relative risk (RR) of congenital anomaly within the circle being calculated by the RR of congenital anomaly outside the circle, in addition to the p-value for each risk area.

We used QGis version 3.10 to create the thematic maps, as well as GeoDa version 1.14 and SaTScan version 9.6 to calculate the spatial statistics.

## Results

Between 2012 and 2021, 28,789,179 live births were registered on the SINASC, of which 18,057 were cases of congenital anomaly, whereby prevalence of congenital anomaly at birth in Brazil was 83.6/10,000 live births. The Southeast region had the highest prevalence of anomaly (98.4/10,000), followed by the Southern region (82.5/10,000), while the Northern region had the lowest prevalence (57.7/10,000).

For Brazil as a whole, the most prevalent types of congenital anomalies in the period were: musculoskeletal system (22.1/10,000 live births), other congenital malformations (11.1/10,000 live births) and congenital foot deformities (9.3/10,000 live births). 

Prevalence of congenital malformations of the circulatory system was higher in the Southeast and Southern regions. Prevalence of nervous system malformations was higher in the Northeast region, when compared to the other regions of the country. Prevalence of congenital anomalies of the digestive system was higher in the Northern region in relation to the other regions of the country (Table 1).

**Table 1:** Congenital anomaly prevalence in (%) per 10,000 live births according to type. Brazil and region, 2012-2021

Anomaly	Prevalence per 10,000 live births (%)					
	North	Northeast	Southeast	South	Midwest	Brazil
Spina bífida	1.5 (2.7)	2.2 (2.8)	2.6 (2.6)	2.4 (2.9)	1.7 (2.5)	2.2 (2.7)
Other congenital malformations of the nervous system	7.3 (12.7)	9.7 (12.4)	8.1 (8.2)	6.2 (7.6)	6.7 (10)	8.1 (9.7)
Congenital malfor-mations of the circu-latory system	2.5 (4.3)	3.3 (4.3)	15.2 (15.4)	9.6 (11.6)	3.8 (5.7)	8.8 (10.5)
Cleft lip and cleft palate	4.9 (8.4)	4.6 (5.9)	5.4 (5.5)	6.8 (8.2)	5.1 (7.6)	5.3 (6.3)
Absence, atresia and stenosis of the small intestine	0.1 (0.2)	0.1 (0.1)	0.3 (0.3)	0.3 (0.3)	0.1 (0.1)	0.2 (0.2)
Other congenital malformations do digestive system	5.1 (8.8)	2.9 (3.7)	4.8 (4.9)	3.5 (4.2)	2.8 (4.2)	4 (4.8)
Undescended testicle	0.5 (0.9)	1.4 (1.8)	2 (2)	1.3 (1.6)	1 (1.5)	1.5 (1.8)
Other malformations of the genitourinary system	2.9 (5)	6.8 (8.7)	7.4 (7.5)	6.9 (8.4)	5 (7.5)	6.5 (7.7)
Congenital deformi-ties of the hip	0.2 (0.3)	0.3 (0.4)	1 (1)	0.3 (0.4)	0.4 (0.6)	0.6 (0.7)
Congenital deformi-ties of the feet	8 (13.9)	10.3 (13.2)	9 (9.1)	9.4 (11.4)	8.8 (13.1)	9.3 (11.1)
Other congenital malformations and deformations of the musculoskeletal system	14.7 (25.5)	22.5 (28.8)	25 (25)	19.9 (24.1)	20.1 (30)	22.1 (26.4)
Other congenital malformations	7.5 (12.9)	10.8 (13.9)	12.9 (13.1)	10.4 (12.6)	9 (13.4)	11.1 (13.3)
Chromosomal ab-normalities not else-where classified	2.2 (3.9)	2.9 (3.7)	4.2 (4.3)	5.1 (6.2)	2.4 (3.7)	3.6 (4.3)
Hemangioma and lymphangioma	0.2 (0.3)	0.2 (0.3)	0.5 (0.5)	0.5 (0.6)	0.2 (0.4)	0.3 (0.4)
Total	57.7 (100)	78.1 (100)	98.4 (100)	82.5 (100)	67.1 (100)	83.6 (100)

**Figure 1:**
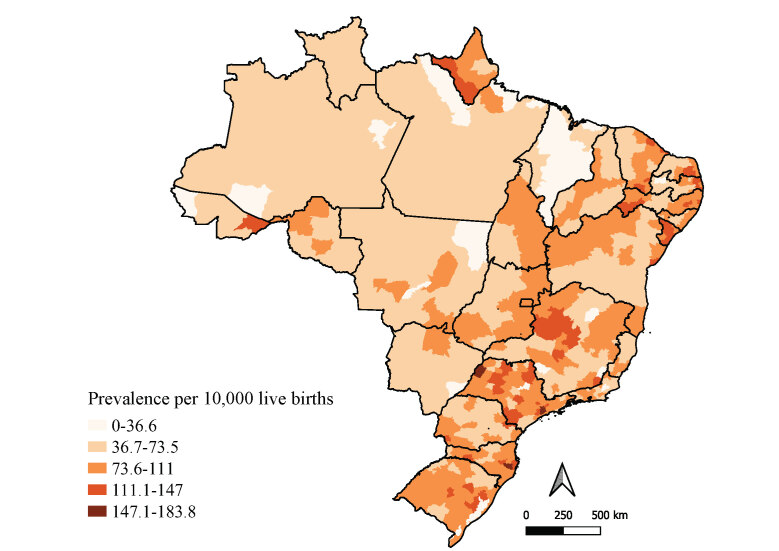
Prevalence of congenital anomalies by immediate geographic region. Brazil, 2012-2021

Regarding spatial analysis of prevalence of anomalies in the immediate geographic regions, high prevalence rates were found to be concentrated in these regions on the Northeast coast, which comprises part of the states of Bahia, Sergipe, Alagoas, Pernambuco, Paraíba and Rio Grande do Norte. The majority of immediate geographic regions with congenital anomaly prevalence above 73.6/10,000 live births were located in the states of Amapá, São Paulo, Santa Catarina and Rio Grande do Sul. In the state of Paraná, the highest anomaly prevalence rate was found in the western region of that state. Immediate geographic regions with the lowest prevalence rates were concentrated in the states of Amazonas, Pará, Roraima and Maranhão (Figure 1).

**Figure 2:**
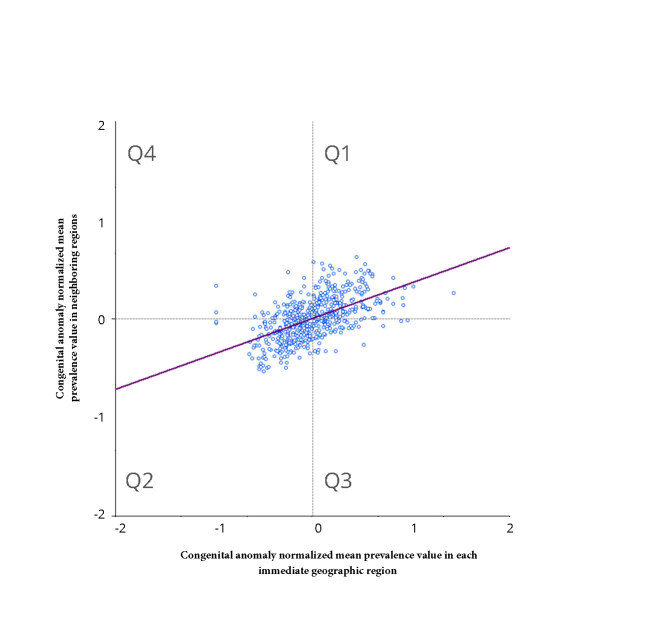
Distribution of congenital anomalies according to Moran’s global index: Brazil, 2012-2021

**Figure 3:**
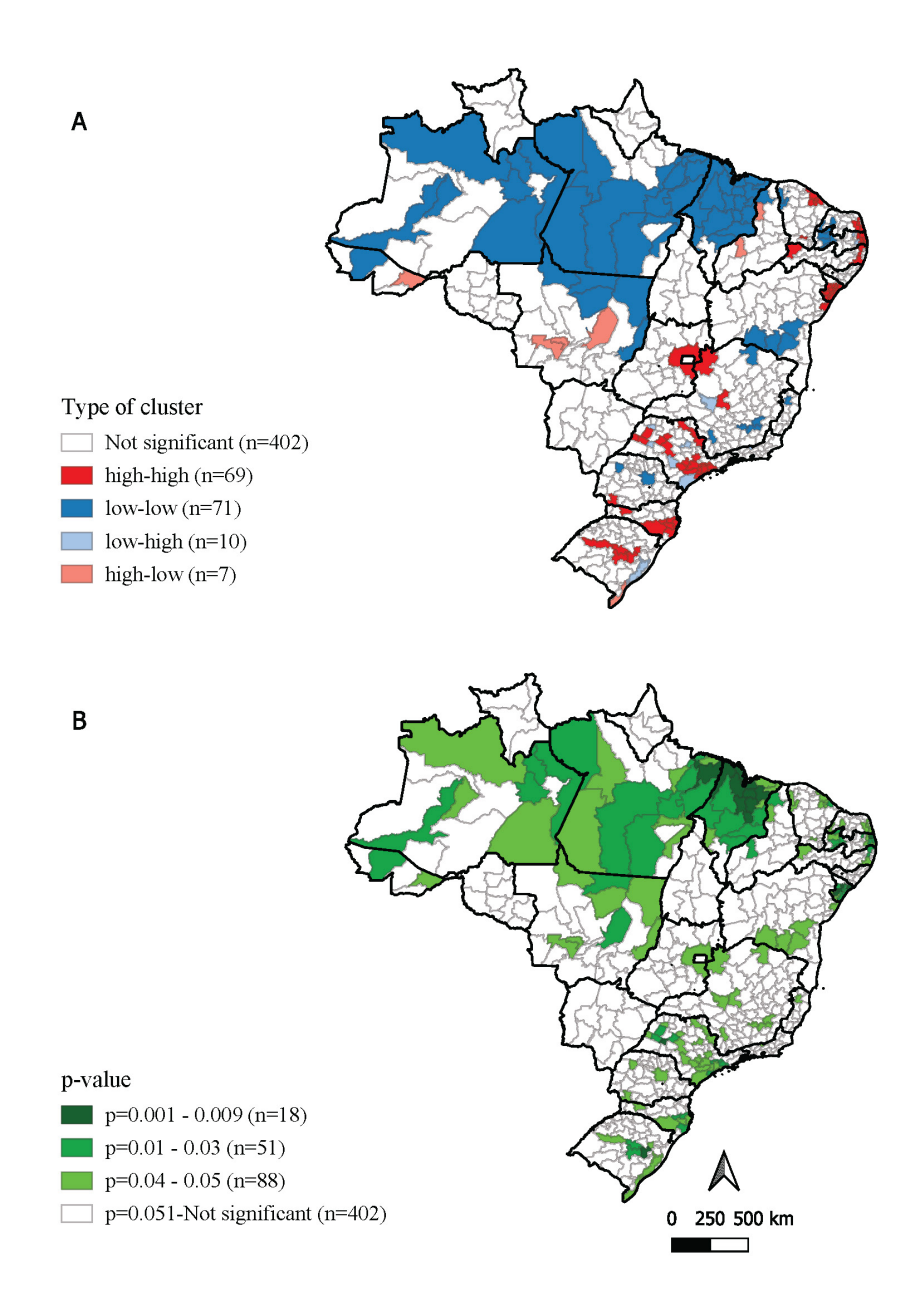
Congenital anomaly clusters, by local indicator of spatial association (A) type of cluster and (B) statistical significance of each type of cluster. Brazil, 2012-2021

The Moran scatterplot showed spatial autocorrelation between prevalence of congenital anomalies at birth, indicating spatial dependence (Moran’s I=0,360; p-value 0.001)) (Figure 2). We identified 69 immediate geographic regions with high-high clustering. Of these, 31 were in the Northeast (Ceará, Bahia, Sergipe, Pernambuco, Paraíba and Rio Grande do Norte); 24 in the Southeast (Minas Gerais and São Paulo); 13 in the South (Paraná, Santa Catarina and Rio Grande do Sul; and 1 in the Midwest (Goiás) (Figure 3).

**Figure 4:**
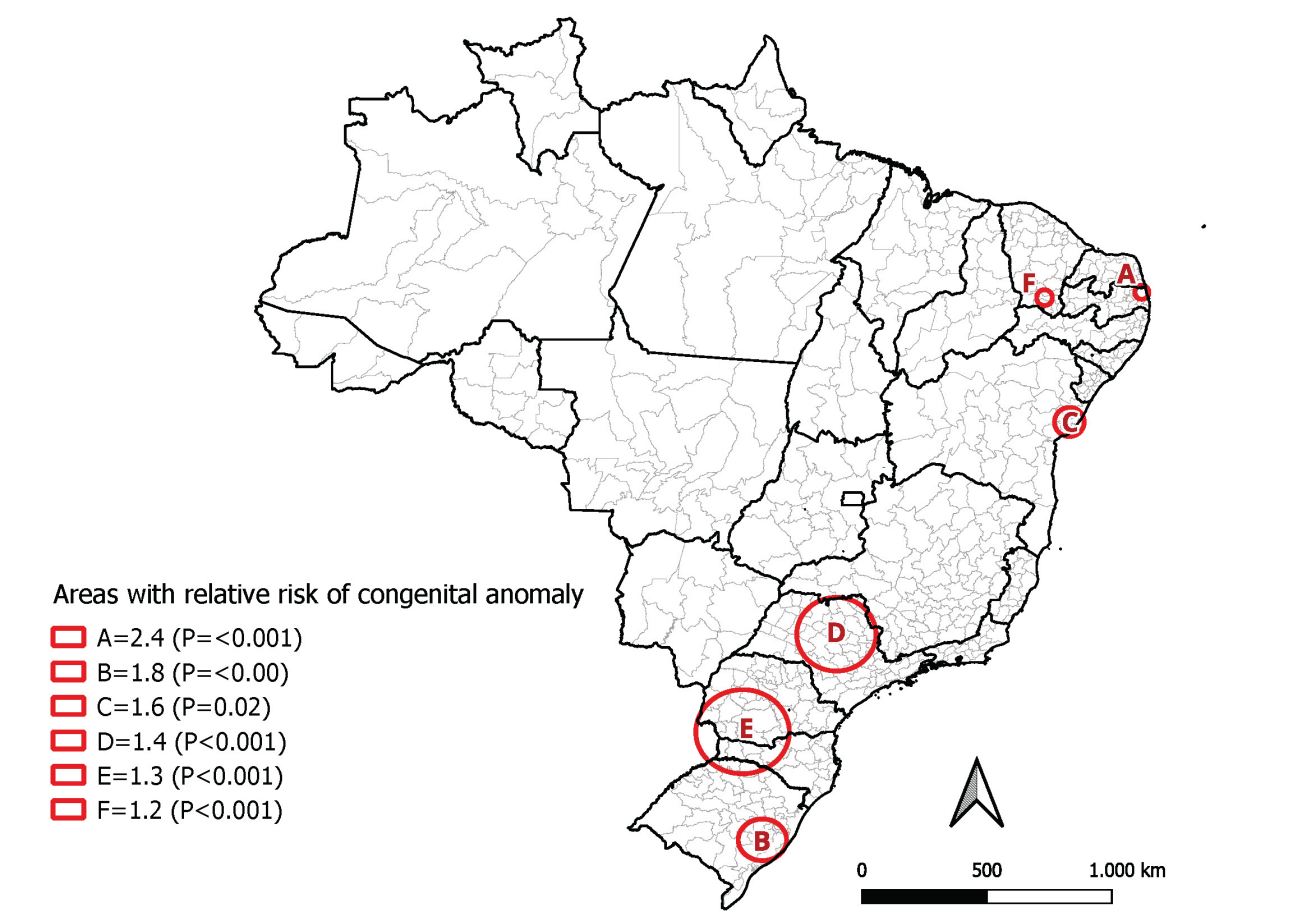
Areas of greater risk of congenital anomalies according to spatial scanning analysis. Brazil, 2012-2021

Six areas with positive relative risk of congenital anomalies were identified by spatial scanning, in immediate geographic regions located in the states of Paraíba, Bahia and Ceará Rio Grande do Sul, São Paulo, Paraná and Santa Catarina (Figure 4).

## Discussion

Differences between the country’s regions were found in the spatial distribution of congenital anomalies, both in terms of total anomalies and specific groups, with higher prevalence in the Southeast and Southern regions, and risk areas in the Northeast, Southeast and Southern regions. Musculoskeletal system anomalies were more common in all regions. 

Incomplete or incorrect SINASC information are limitations inherent to all research that uses data collected by health services as part of their routine work. As some anomalies are diagnosed at other stages of development, cases may be underrecorded on our data source, including stillbirths with anomalies, which are not recorded on the SINASC. In most Brazilian municipalities, coverage of information on live births held on the SINASC is adequate (13).

Greater frequency of musculoskeletal system anomalies has also been found by other studies in Brazil and in other Latin American countries (14,15). Ease of diagnosis through physical examination performed at birth may explain higher prevalence of musculoskeletal system anomalies, since live birth certificates, which are input to the SINASC, are issued within a few hours after birth. 

This could explain why the circulatory system is the fourth most affected system by congenital malformations among live births. These anomalies are more difficult to diagnose and can manifest themselves at other stages of development. In Europe, high-quality sources are used to collect data on live birth anomalies and fetal deaths in terminated pregnancies. Among Europeans, congenital heart defects are the most prevalent anomalies, with a rate of 65/10,000 live births (16). In Brazil, circulatory system anomalies are the main category of congenital anomaly as a cause of infant mortality. This important rate is not recorded on the SINASC, but rather is only recorded on the Mortality Information System (17).

The Northeast region concentrated the highest number of areas at risk for congenital anomalies. Some factors may explain this phenomenon, such as the Zika virus outbreak, which occurred in 2015, inbreeding and the low human development index in the region (18,19). The results of this study showed that the Northeast was the region with the highest prevalence of anomalies in the group of “other congenital malformations of the nervous system”, which includes microcephaly. This greater occurrence can be explained by the fact that the region was considered the epicenter of the congenital Zika syndrome epidemic in Brazil (20). The Northeast region has one of the highest rates of inbreeding in Brazil, this being a risk factor for the occurrence of autosomal recessive disorders. These disorders are associated with higher prevalence of congenital anomalies and clusters of cases of rare genetic diseases (21). The Northeast has the highest number of clusters of genetic disorders in Brazil, but surveillance of them is weak and scarce (22). There is therefore an urgent need to develop public health policies aimed at epidemiological surveillance of congenital defects and rare genetic disorders in the Northeast region. 

Although inequalities between Brazilian regions have decreased, the Northeast and Northern regions still have the lowest human development indices in Brazil and a larger portion of the population faces poverty and has less access to health services (23). This indicator summarizes three basic dimensions of human development: income, education and health (24). Considering that around 30% of congenital anomalies have an environmental or multifactorial cause and many still have an unknown etiology, these dimensions can directly and indirectly impact prevalence of congenital anomalies, given that 94% of serious birth defects occur in low- and middle-income countries (25).

Other studies have also reported higher prevalence of congenital anomalies in the Southern and Southeast regions compared to other regions of Brazil, particularly the Northern region, (26,17) which may be related to the greater concentration of medical services and better recording on the SINASC in those regions. The municipality of São Paulo, for example, has been developing continuing education actions aimed at improving diagnosis of congenital anomalies, their registration on live birth certificates DNV and recording on the SINASC, in partnership with the Medical Genetics Center of the Universidade Federal de São Paulo since 2005. These efforts regarding continuing education and continuous monitoring have led to better SINASC data quality, with a reduction in unknown or missing information and an increase in reporting of congenital anomaly cases (27). The Northern region had the lowest prevalence of congenital anomalies. This may be related to reduced health service accessibility, due to geographic isolation and long distances from specialized medical centers, contrary to what occurs in the Southeast region.

Another area of risk of congenital anomalies was found in the west of the states of Paraná and Santa Catarina. These states border Paraguay and Argentina, countries where the policy on commercialization and consumption of products for agricultural cultivation is less restrictive than in Brazil. Easy access to these products in neighboring countries may be favoring their use in Brazil’s border regions. Given the teratogenic effect of these substances, reported by other research, (28,29) the need exists to conduct new studies to investigate the relationship between use of pesticides and prevalence of congenital anomalies in Brazilian border regions. 

We found heterogeneity in the spatial distribution of congenital anomalies in Brazil, with risk areas in the Northeast, Southeast and Southern regions. The Northeast had the highest number of areas with high relative risk of anomalies. In a scenario of limited resources, areas at high risk need to be prioritized regarding implementation of public policies aimed at preventing anomalies.

Actions aimed at improving the filling out of SINASC data are essential for good quality information that can increase the capacity of local health service management to plan and evaluate maternal and child health care policies that include primary prevention of congenital anomalies. We suggest that further research be carried out focusing on the correlation between socio-environmental conditions and the occurrence of congenital anomalies in Brazil, especially in regions with high risk.
