# Supplementary material for: Spatial analysis of risk areas of congenital anomalies in Brazil, 2012-2021
Source: Epidemiol Serv Saude. 2025 May 9;34:e20250240. doi: 10.1590/S2237-96222025v34e20250240.en (PMC12064163; doi:10.1590/S2237-96222025v34e20250240.en)
Supplement: Translation

# Translation: Análise espacial de áreas de risco de anomalias congênitas no Brasil, 2012-2021

**DOI:** 10.1590/S2237-96222025v34e20250240.pt

**Authors:** Suzana de Souza, Clarissa Gutierrez Carvalho, Lavinia Schuler-Faccini

**Affiliations:** 1 Universidade Federal do Rio Grande do Sul, Programa de Pós-graduação em Saúde da Criança e do Adolescente, Porto Alegre, RS, Brasil; 2 Secretaria Estadual de Saúde do Rio Grande do Sul, Assessoria de Gestão e Planejamento, Porto Alegre, RS, Brasil; 3 Hospital de Clínicas de Porto Alegre, Serviço de Neonatologia, Porto Alegre, RS, Brasil; 4 Hospital de Clínicas de Porto Alegre, Serviço de Genética Médica, Porto Alegre, RS, Brasil

**Keywords:** Anormalidades Congênitas, Risco, Recém-nascido, Análise Espacial, Meio Ambiente e Saúde Pública

## Abstract

**Objetivo:**

Identificar áreas de risco de anomalias congênitas no Brasil no período 2012-2021.

**Método:**

Trata-se de análise de série temporal utilizando dados do Sistema de Informação Sobre Nascidos Vivos. A prevalência de anomalia no período foi calculada por região geográfica imediata. A análise espacial foi realizada pelo cálculo do índice de Moran global e local e varredura espacial, com cálculo do risco relativo (RR) e p-valor, para as áreas de risco.

**Resultados:**

Áreas de maior risco de anomalias foram identificadas nas regiões Nordeste, Sudeste e Sul. O Nordeste apresentou maior número de aglomerados de risco (n=31) e maior prevalência de anomalias do sistema nervoso em relação às outras regiões (9,7/10.000 nascidos vivos). O maior risco de anomalias em relação às demais áreas foi observado na Paraíba (RR 2,4; p-valor<0,001).

**Conclusão:**

Disparidades na distribuição de anomalias congênitas no Brasil foram identificadas, com áreas de risco nas regiões Nordeste, Sudeste e Sul.

## Introdução

Anomalia congênita pode ser definida como qualquer alteração estrutural, funcional ou metabólica que afeta o desenvolvimento embrionário e/ou fetal. Fatores genéticos, ambientais ou uma combinação de ambos (multifatoriais) estão em sua etiologia, embora, em muitos casos, não seja possível identificá-la (1). Mundialmente, entre 3% a 6% das crianças nasceram com algum tipo de anomalia em 2022 (2). Esta foi uma das principais causas de mortalidade e incapacidade entre as crianças nos países industrializados (3). Com a melhora das condições sanitárias e nutricionais e a redução da mortalidade infantil por doenças infecciosas e parasitárias, as anomalias congênitas passaram a ser a primeira causa de óbito infantil no Brasil, respondendo por 22% dos os óbitos em menores de um ano (2). 

O Brasil apresenta grande variabilidade genética e diversidade de fatores ambientais incluindo variáveis socioeconômicas, culturais, raciais e étnicas que podem impactar a distribuição de doenças e agravos para a saúde (4). Saber como as anomalias congênitas estão distribuídas no espaço possibilita o reconhecimento de aglomerados. Isso pode ser um ponto de partida para a identificação de suscetibilidade genética e de fatores ambientais associados à ocorrência de anomalias congênitas em regiões com alta prevalência.

A prevenção primária das anomalias congênitas baseia-se no controle ambiental dos fatores de risco. Realizar estudos para identificar essas áreas é de fundamental importância, uma vez que o planejamento das ações em saúde pode ser mais bem direcionado para fatores de risco modificáveis. Conhecer a distribuição espacial das anomalias congênitas também pode contribuir com a formulação de políticas públicas na perspectiva da prevenção terciária. Esta tem como objetivo evitar as complicações das anomalias congênitas através da reabilitação e da correção adequadas (2). A epidemiologia espacial possibilita uma visão antecipada do risco coletivo. Essa antecipação é importante em termos de saúde pública, já que os processos endêmicos e as intervenções de saúde pública nos vários grupos sociais devem ser analisados a partir de uma perspectiva ecológica (5). 

Este estudo objetivou identificar áreas de risco de anomalias congênitas no Brasil.

## MÉTODOS

### Delineamento e contexto

Trata-se de estudo de série temporal que considerou como unidade de análise as regiões geográficas imediatas do Brasil, sendo: 188 no Nordeste; 160 no Sudeste; 96 no Sul; 64 no Norte; e 52 no Centro-Oeste. Tais regiões, refletem a organização do espaço produtivo e polos de desenvolvimento e estrutura urbano-industrial (6).

### Controle de viés

Optou-se por essa unidade no presente estudo para corrigir flutuações aleatórias e proporcionar melhor estabilidade, principalmente em áreas com populações muito pequenas.

### Participantes

A população de estudo constitui-se de todos os nascidos vivos com anomalia congênita registrada na declaração de nascido vivo no Brasil entre 2012 e 2021.

### Fonte de dados

Os dados de todos os nascidos vivos, distribuídos nas regiões políticas e nas regiões geográficas imediatas, foram extraídos do Sistema de Informação Sobre Nascidos Vivos (Sinasc) no TabNet, disponível no sítio eletrônico do Departamento de Informática do Sistema Único de Saúde.

### Variáveis

Os dados foram extraídos do Sinasc de acordo com o tipo de anomalia, considerando os seguintes códigos do capítulo 17, Malformações congênitas, deformidades e anomalias cromossômicas (Q00-Q99), da Classificação Estatística Internacional de Doenças e Problemas Relacionados à Saúde 10ª versão: anomalias congênitas do sistema nervoso (Q00-Q07); espinha bífida (Q05); anomalias congênitas do aparelho circulatório (Q20-Q28); fenda labial e palatina (Q35-Q37); ausência de atresia e estenose do intestino delgado (Q38-Q41); aparelho digestivo (Q48-Q45); testículo não descido (Q53); malformação do aparelho urinário (Q60-Q64); deformidades congênitas do quadril (Q65); deformidades congênitas dos pés (Q66); malformação do aparelho osteomuscular (Q65-Q79); outras malformações congênitas (Q80-Q89); e anomalias cromossômicas (Q90-Q99).

### Tamanho do estudo e mensuração

Os dados foram organizados em uma planilha de Excel. Calculou-se a prevalência de acordo com o tipo de anomalia congênita no período total de estudo (2012 a 2021) para as cinco regiões do país. Para realizar o cálculo da prevalência, a soma dos casos de anomalias congênitas da região foi incluída no numerador e, no denominador, a soma do total de nascidos vivos da mesma região, multiplicado por 10.000. Adicionalmente, realizou-se análise descritiva da distribuição percentual dos registros, segundo tipo de anomalia e região. Para esse cálculo, o tipo de anomalia congênita foi tomado no numerador e, no denominador, a soma de todos os tipos de anomalia, multiplicado por cem.

### Métodos estatísticos

Para a análise estatística espacial, construiu-se mapa temático das prevalências, categorizadas em quintis da distribuição. Empregou-se o Datum SIRGAS 2000 como sistema de projeção de coordenadas geográficas, sistema de referência geocêntrico para as Américas, oficialmente adotado no Brasil (7). Os mapas do Brasil foram obtidos – em formato vetorial (shapefile) – no site do Instituto Brasileiro de Geografia e Estatística (8). 

A identificação de áreas de risco foi realizada mediante a análise de autocorrelação espacial e a análise de varredura espacial. A análise de autocorrelação espacial foi feita por meio do cálculo do índice de global de Moran (I de Moran) e o indicador local de associação espacial (LISA). Foi empregada a contiguidade do tipo “rainha” como matriz de pesos espaciais, que considera os vértices (nós) com ligação pontual (9). 

O diagrama de dispersão de Moran foi utilizado para a visualização da autocorrelação espacial. A análise foi realizada comparando a prevalência de anomalia em cada região geográfica imediata com as prevalências das regiões vizinhas. Os dados foram apresentados em quatro quadrantes: alto-alto, baixo-baixo, alto-baixo e baixo-alto. Foram considerados aglomerados de risco (alto-alto) os dados que se concentraram no quadrante 1, onde a prevalência de anomalia congênita em cada polígono e o valor médio da prevalência nos polígonos vizinhos foi maior que a média global. O diagrama foi construído com base nos valores normalizados da prevalência de anomalia congênita, ou seja, os valores originais subtraídos do valor médio global, divididos pelo desvio-padrão. O resultado da associação foi representado pelo I de Moran, que apresenta valores entre 0 e 1 (10).

O LISA foi empregado, complementarmente, para analisar a associação espacial local. Ele produziu o grau de autocorrelação em cada região geográfica imediata e permitiu uma visualização geográfica mais adequada do grau de concentração de anomalias congênitas. O mapa de agrupamento foi obtido por meio da combinação das informações do diagrama de dispersão de Moran com as estatísticas LISA (11).

Utilizou-se a técnica de análise de varredura espacial (12) para identificar áreas de risco de anomalias congênitas, por meio da inserção de círculo virtual em torno do centro das regiões geográficas imediatas e cálculo da prevalência de ocorrência de anomalias. Foi considerado agrupamento de risco quando a prevalência observada foi maior que a esperada, ou seja, o número anomalias em cada área não foi proporcional ao tamanho da sua população. Para a varredura espacial, foi utilizado o modelo discreto de Poisson, e a análise foi condicionada ao número total de casos observados com tamanho máximo de agrupamento igual a 50% da população exposta. As análises foram de variação puramente espacial, sendo calculado o risco relativo (RR) de anomalia congênita dentro do círculo, pelo RR de anomalia congênita fora do círculo, além do p-valor para cada área de risco.

O programa utilizado para a confecção dos mapas temáticos foi o QGis versão 3.10. Os programas utilizados na estatística espacial foram o GeoDa versão 1.14 e o SaTScan versão 9.6.

## Resultados

De 2012 a 2021, foram registrados 28.789.179 nascidos vivos no Sinasc, dos quais 18.057 eram casos de anomalia congênita, com prevalência de anomalia congênita ao nascimento no Brasil de 83,6/10.000 nascidos vivos. A região Sudeste apresentou a maior prevalência de anomalia (98,4/10.000), seguida da região Sul (82,5/10.000), enquanto a região Norte apresentou a menor prevalência (57,7/10.000).

No país, os tipos de anomalias congênitas mais prevalente no período foram: aparelho osteomuscular (22,1/10.000 nascidos vivos), outras malformações congênitas (11,1/10.000 nascidos vivos) e deformidades congênitas do pé (9,3/10.000 nascidos vivos). 

A prevalência de anomalias congênitas do aparelho circulatório foi mais elevada nas regiões Sudeste e Sul. No Nordeste, a prevalência das anomalias do sistema nervoso foi maior, quando comparada as outras regiões. No Norte, a prevalência de anomalias congênitas do aparelho digestivo foi maior do que as encontradas nas demais regiões (Tabela 1).

**Tabela 1:** Prevalência em (%) por 10.000 nascidos vivos de anomalia congênita de acordo com o tipo. Brasil e regiões, 2012-2021

Anomalia	Prevalência por 10.000 nascidos vivos (%)					
	Norte	Nordeste	Sudeste	Sul	Centro-Oeste	Brasil
Espinha bífida	1,5 (2,7)	2,2 (2,8)	2,6 (2,6)	2,4 (2,9)	1,7 (2,5)	2,2 (2,7)
Outras malforma-ções congênitas do sistema nervoso	7,3 (12,7)	9,7 (12,4)	8,1 (8,2)	6,2 (7,6)	6,7 (10)	8,1 (9,7)
Malformações con-gênitas do aparelho circulatório	2,5 (4,3)	3,3 (4,3)	15,2 (15,4)	9,6 (11,6)	3,8 (5,7)	8,8 (10,5)
Fenda labial e fenda palatina	4,9 (8,4)	4,6 (5,9)	5,4 (5,5)	6,8 (8,2)	5,1 (7,6)	5,3 (6,3)
Ausência, atresia e estenose do intestino delgado	0,1 (0,2)	0,1 (0,1)	0,3 (0,3)	0,3 (0,3)	0,1 (0,1)	0,2 (0,2)
Outras malformações congênitas do aparelho digestivo	5,1 (8,8)	2,9 (3,7)	4,8 (4,9)	3,5 (4,2)	2,8 (4,2)	4 (4,8)
Testículo não descido	0,5 (0,9)	1,4 (1,8)	2 (2)	1,3 (1,6)	1 (1,5)	1,5 (1,8)
Outras malformações do aparelho geniturinário	2,9 (5)	6,8 (8,7)	7,4 (7,5)	6,9 (8,4)	5 (7,5)	6,5 (7,7)
Deformidades congênitas do quadril	0,2 (0,3)	0,3 (0,4)	1 (1)	0,3 (0,4)	0,4 (0,6)	0,6 (0,7)
Deformidades con-gênitas dos pés	8 (13,9)	10,3 (13,2)	9 (9,1)	9,4 (11,4)	8,8 (13,1)	9,3 (11,1)
Outras malformações e deformidades congênitas do aparelho osteomuscular	14,7 (25,5)	22,5 (28,8)	25 (25)	19,9 (24,1)	20,1 (30)	22,1 (26,4)
Outras malformações congênitas	7,5 (12,9)	10,8 (13,9)	12,9 (13,1)	10,4 (12,6)	9 (13,4)	11,1 (13,3)
Anomalias cromossômicas não codifi-cadas em outra parte	2,2 (3,9)	2,9 (3,7)	4,2 (4,3)	5,1 (6,2)	2,4 (3,7)	3,6 (4,3)
Hemangioma e linfangioma	0,2 (0,3)	0,2 (0,3)	0,5 (0,5)	0,5 (0,6)	0,2 (0,4)	0,3 (0,4)
Total	57,7 (100)	78,1 (100)	98,4 (100)	82,5 (100)	67,1 (100)	83,6 (100)

Com relação à análise espacial da prevalência de anomalias aplicadas nas regiões geográficas imediatas, a concentração de regiões com prevalências elevadas foi observada no litoral do Nordeste, que compreende parte da Bahia, de Sergipe, de Alagoas, de Pernambuco, da Paraíba e do Rio Grande do Norte. A maioria das regiões geográficas imediatas apresenta prevalência de anomalia congênita acima de 73,6/10.000 nascidos vivos no Amapá, em São Paulo, em Santa Catarina e no Rio Grande do Sul. No Paraná, as maiores prevalências de anomalia foram observadas na região oeste do estado. O Amazonas, o Pará, Roraima e o Maranhão concentraram as regiões geográficas imediatas com as menores prevalências (Figura 1).

**Figura 1:** Prevalência de anomalias congênitas por região geográfica imediata. Brasil, 2012-2021

O diagrama de dispersão de Moran mostrou autocorrelação espacial entre as prevalências ao nascimento de anomalias congênitas, indicando dependência espacial (Moran I= 0,360; p-valor 0,001) (Figura 2). Foram identificadas 69 regiões geográficas imediatas envolvidas em agrupamento alto-alto. Destas, 31 ficam no Nordeste (Ceará, Bahia, Sergipe, Pernambuco, Paraíba e Rio Grande do Norte); 24, no Sudeste (Minas Gerais e São Paulo); 13, no Sul (Paraná, Santa Catarina e Rio Grande do Sul; e 1, no Centro-Oeste (Goiás) (Figura 3).

**Figura 2:** Distribuição de anomalias congênitas segundo índice de Moran global. Brasil, 2012-2021

**Figura 3:** Aglomerados de anomalias congênitas, segundo o indicador local de associação espacial (A) tipo de aglomerado, (B) significância estatística de cada tipo de aglomerado. Brasil, 2012-2021

Seis áreas com risco relativo positivo de anomalias congênitas foram identificadas na varredura espacial, em regiões geográficas imediatas localizadas na Paraíba, na Bahia e no Ceará, Rio Grande do Sul, São Paulo, Paraná e Santa Catarina (Figura 4).

**Figura 4:** Áreas de maior risco de anomalias congênitas segundo análise de varredura espacial. Brasil, 2012-2021

## DISCUSSÃO

A distribuição espacial das anomalias congênitas apresentou diferenças entre regiões tanto no total das anomalias quanto a grupos específicos, com prevalência maior nas regiões Sudeste e Sul e áreas de risco nas regiões Nordeste, Sudeste e Sul. Anomalias do sistema osteomuscular foram mais comuns em todas as regiões. 

A incompletude ou o erro das informações provenientes dos Sinasc são limitações inerentes a toda pesquisa que utiliza dados coletados pelos serviços de saúde na rotina do trabalho. Como algumas anomalias são diagnosticadas em outros estágios do desenvolvimento, nossa fonte de dados pode conter sub-registros desses casos, inclusive com relação aos natimortos com anomalia, que não são registrados no Sinasc. Em grande parte dos municípios brasileiros, a cobertura das informações de nascidos vivos, apresentada no Sinasc, é adequada (13).

A maior frequência de anomalias do sistema osteomuscular também foi observada em outros estudos no Brasil e em outros países da América Latina (14,15). A facilidade de diagnóstico ao exame físico realizado no momento do nascimento pode explicar a maior prevalência de anomalias do sistema osteomuscular, uma vez que a declaração de nascidos vivos, que alimenta o Sinasc, é preenchida em poucas horas depois do nascimento. 

Isso poderia explicar por que as anomalias congênitas do aparelho circulatório representam apenas o quarto sistema mais afetado em nascidos vivos. Essas anomalias são mais difíceis de serem diagnosticadas e podem se manifestar em outros estágios do desenvolvimento. A população europeia utiliza fontes de alta qualidade para coletar dados sobre anomalias em nascidos vivos e mortes fetais em gestações interrompidas. Nessa população, as cardiopatias congênitas são as anomalias mais prevalentes, com uma taxa de 65/10.000 nascidos vivos (16). No Brasil, as anomalias do sistema circulatório são a principal categoria de anomalia congênita como causa de mortalidade infantil. Essa importante proporção não aparece registrada no Sinasc, mas apenas no Sistema de Mortalidade (17).

A região Nordeste concentrou o maior número de áreas de risco de anomalias congênitas. Alguns fatores podem explicar esse fenômeno, como o surto de vírus Zika, que ocorreu a partir de 2015, a consanguinidade e o baixo índice de desenvolvimento humano na região (18,19). Os resultados deste estudo mostraram que o Nordeste foi a região que apresentou maior nível de prevalência de anomalias do grupo “outras anomalias do sistema nervoso central”, que inclui a microcefalia. Essa maior ocorrência pode ser justificada pelo fato de a região ter sido considerada o epicentro da epidemia de síndrome congênita do Zika no país (20). Tal região apresenta uma das maiores taxas de endogamia no país, fator de risco para ocorrência de doenças autossômicas recessivas. Essas doenças estão associadas à maior prevalência de anomalias congênitas e de aglomeração de casos de doenças genéticas raras (21). O Nordeste apresenta o maior número de rumores de aglomerados de doenças genéticas no Brasil, porém sua vigilância é frágil e escassa (22). Isso torna urgente a elaboração de políticas públicas de saúde voltadas para a vigilância epidemiológica dos defeitos congênitos e das doenças genéticas raras nessa região. 

Apesar de as desigualdades entre as regiões brasileiras terem diminuído, as regiões Nordeste e Norte ainda concentram os menores índices de desenvolvimento humano do Brasil e maior parcela da população encontra-se em situação de pobreza e possui menor acesso aos serviços de saúde (23). Tal indicador resume três dimensões básicas do desenvolvimento humano: renda, educação e saúde (24). Considerando que cerca de 30% das anomalias congênitas têm causa ambiental ou multifatorial e muitas ainda têm etiologia desconhecida, essas dimensões podem impactar direta e indiretamente a prevalência de anomalias congênitas, já que 94% dos defeitos congênitos graves ocorrem em países de baixa e média renda (25).

Outros estudos também mostraram maior prevalência de anomalias congênitas nas regiões Sul e Sudeste em relação às demais regiões, particularmente a região Norte, (26,17) o que pode ter relação com a maior concentração de serviços médicos e melhor registro no Sinasc. O município de São Paulo, por exemplo, desenvolve ações de educação permanente voltadas ao aprimoramento do diagnóstico das anomalias congênitas, seu registro na DNV e no Sinasc, em parceria com o Centro de Genética Médica da Universidade Federal de São Paulo desde 2005. Esse trabalho de capacitação e monitoramento contínuos levou à melhor qualidade dos dados da base do Sinasc, observando-se o declínio das informações ignoradas ou ausentes e o aumento da notificação de casos anomalias congênitas (27). A região Norte apresentou a menor prevalência de anomalias congênitas. Isso pode estar relacionado à menor acessibilidade aos serviços de saúde, devido ao isolamento geográfico e às grandes distâncias dos centros médicos especializados, ao contrário do que ocorre na região Sudeste.

Outra área de risco de anomalia congênita foi observada no oeste do Paraná e de Santa Catarina. Esses estados fazem fronteira com o Paraguai e a Argentina, países onde a política de comercialização e consumo de produtos para o cultivo agrícola é menos restritiva do que no Brasil. O fácil acesso a esses produtos nos países vizinhos pode estar favorecendo seu uso nas regiões fronteiriças do Brasil. Dado o efeito teratogênico dessas substâncias, observados em outras pesquisas, (28,29) torna-se necessária a realização de novos estudos que investigam a relação entre o uso de agrotóxicos e a prevalência de anomalias congênitas nas regiões fronteiriças do Brasil. 

Observou-se heterogeneidade na distribuição espacial das anomalias congênitas no Brasil, com áreas de risco nas regiões Nordeste, Sudeste e Sul. O Nordeste apresentou o maior número de áreas com risco relativo elevado de anomalias. Em um cenário de recursos limitados, as áreas com risco elevado precisam ser priorizadas na implementação de políticas públicas que visem a prevenção de anomalias.

Ações que visem o aprimoramento no preenchimento dos dados do Sinasc são fundamentais para a qualificação das informações que podem aumentar a capacidade dos níveis locais de planejar e avaliar as políticas destinadas à saúde materno infantil que contemplem a prevenção primária das anomalias congênitas. Sugere-se a realização de novas pesquisas com foco na correlação entre condicionantes socioambientais e a ocorrência de anomalias congênitas no Brasil, sobretudo nas regiões com risco elevado.
